# Autosomal dominant Best disease with an unusual electrooculographic light rise and risk of angle-closure glaucoma: a clinical and molecular genetic study

**Published:** 2011-08-23

**Authors:** Sancy Low, Alice E. Davidson, Graham E. Holder, Chris R. Hogg, Shomi S. Bhattacharya, Graeme C. Black, Paul J. Foster, Andrew R. Webster

**Affiliations:** 1 NIHR Biomedical Research Centre for Ophthalmology, Moorfields Eye Hospital and UCL Institute of Ophthalmology, London, UK; 2 Genetic Medicine, The University of Manchester, Manchester Academic Health Science Centre, Central Manchester University Hospitals NHS Foundation Trust, Manchester, UK

## Abstract

**Purpose:**

To describe the clinical and molecular characteristics of two families with autosomal dominant Best disease and atypical electrooculography (EOG).

**Methods:**

Four affected individuals from two families were ascertained. Detailed ophthalmic examinations, refraction, and biometry (anterior chamber depth [ACD] and axial length [AL]), gonioscopy, optical coherence tomography of the anterior segment and retina, retinal imaging, and electrophysiological assessment were performed. Arden ratios from EOG testing were calculated by direct measurement of the light peak to dark trough amplitudes. Mutations in bestrophin 1 (*BEST1*) were identified by bidirectional Sanger sequencing. In family 1, segregation of *BEST1* alleles was performed by assaying four microsatellite markers (D11S935, D11S4102, D11S987, and D11S4162) that flank *BEST1*.

**Results:**

The proband from family 1 (three of four siblings affected with Best disease) was 42 years old with bilateral macular vitelliform lesions, advanced angle closure glaucoma (ACG), a normal electroretinogram, and no EOG light rise. Her 44-year-old brother had similar fundus appearances and an EOG light rise of 170%. Their 48-year-old sister had a normal left fundus, whereas the right fundus showed a vitelliform lesion and subretinal thickening. There was no EOG light rise detectable from either eye. Mutation analysis of *BEST1* showed all affected siblings to be heterozygous for a missense mutation, c.914T>C, p.Phe305Ser. Their unaffected sister had an EOG light rise of 200%, a normal fundus appearance, and did not harbor the *BEST1* mutation. Haplotype analysis of family 1 showed that the affected brother with the 170% EOG light rise had inherited the same nondiseased parental *BEST1* allele as his unaffected sister. The other two affected sisters with undetectable EOG light rises shared a different nondiseased parental *BEST1* allele. An unrelated 53-year-old female carrying the same c.914T>C, p.Phe305Ser mutation showed typical features of Best disease and an EOG light rise of 180%. All four siblings from family 1 had shorter axial biometry (ACD range 2.06–2.74 mm; AL range 20.46–22.60 mm) than the normal population, contributing to their risk of ACG development. Proband 2 had deeper ACDs (2.83 mm OD and 2.85 mm OS), but similar ALs (21.52 mm OD and 21.42 mm OS) compared to family 1. She had no gonioscopic evidence of angle closure.

**Conclusions:**

A near normal EOG light rise is uncommon in molecularly confirmed Best disease, and in the present report is associated with the same mutation in two families, suggesting a specific role for this amino acid in the retinal pigment epithelium dysfunction associated with this disorder. Haplotype analysis in family 1 was consistent with an effect of the nondisease allele in mediating the presence of an EOG light rise. Clinical assessment of ACG risk is recommended for *BEST1* mutation carriers and their first degree relatives.

## Introduction

Best disease (OMIM 153700) is an autosomal dominant macular dystrophy characterized by vitelliform macular lesions, a normal electroretinogram and reduction of the electooculogram (EOG) light rise. Best disease was first linked to bestrophin-1 (*BEST1*; *VMD2*) in 1998 [1,2]. Mutations in *BEST1* have since been found to be causative of other distinctive retinal dystrophies, including autosomal dominant vitreoretinochoroidopathy (ADVIRC; OMIM 193220) [3–7], autosomal recessive bestrophinopathy (ARB; OMIM 611809) [8–10], adult vitelliform macular dystrophy [11,12], and more recently, what was described as “concentric retinitis pigmentosa” [13]. A severely reduced EOG light rise remains the most sensitive clinical test for the diagnosis of all bestrophinopathies [14].

The *BEST1* gene encodes bestrophin-1, a transmembrane protein located in the basolateral membrane of the retinal pigment epithelium (RPE) [15–18]. Current available evidence suggests that the EOG light rise results from chloride conductance across the basolateral membrane of the RPE [19]. Exogenous expression of bestrophin-1 produces a chloride ion conductance, and bestrophin-1 may function as a calcium-sensitive chloride channel directly responsible for generating the light rise of the EOG [20]. It has been recently suggested that bestrophin-1 may indirectly modulate the light rise component of the EOG in vivo [20–24].

The clinical, electrophysiological, and molecular genetic findings in two families with Best disease are presented in this report. Both families carry the same mutation in the *BEST1* gene, and each family had one individual unexpectedly showing a good EOG light rise.

## Methods

Five subjects (aged 42 to 53) from two families were recruited from clinics at Moorfields Eye Hospital. Slit-lamp examinations, including dark-room gonioscopy performed with a 1 mm slit-beam with a one-mirror Goldmann-style lens (Magnaview goniolens, Ocular Instruments, WA), applanation tonometry, and disc and fundus examinations with an aspheric lens were conducted. Autorefraction (Humphrey autorefractor; Carl Zeiss Meditec, Jena, Germany) and axial biometry measurements of anterior chamber depth (ACD) and axial length (AL) were performed (IOLMaster, Carl Zeiss Meditec, Dublin, CA). Fundus imaging included: autofluorescence (HRA 2; Heidelberg Engineering, Heidelberg, Germany), optical coherence tomography (3D-OCT; Topcon, Paramus, New Jersey), and fundus color photography (Topcon). Anterior segment OCT (AS-OCT) was performed under dark-room conditions when angle closure or secondary glaucoma was suspected (Visante; Carl Zeiss Meditec, Dublin, CA).

Standard electrophysiology was performed that incorporated the relevant ISCEV standards [25–27]. In short, EOG testing was performed with surface electrodes placed at the medial and lateral canthi of each eye. Thirty degree eye movements were recorded for 10 s each minute during 15 min dark adaptation, followed by a further 10–15 min in full-field (Ganzfeld) light adaptation (100 cd/m^2^). The amplitude of the dark trough and light peak were directly measured and Arden ratios calculated [25]. The EOG recordings from these two families were independently measured by three observers (SL, GEH, and CH) and average values used. Normative data were provided by 30 individuals aged between 30 and 60 years of age with no ocular abnormalities. One eye from each subject was randomly selected and used to establish the normative data.

Genomic DNA was extracted from peripheral blood with the Qiagen Blood DNA extraction kit (Qiagen, Crawley, UK). Bidirectional Sanger sequencing analysis of the entire coding regions of *BEST1*, including the splice donor and acceptor sites of the gene, was performed with BigDye 3.1 (Applied Biosystems, Warrington, UK). Primer sequences and annealing temperatures are shown in Table 1. The cDNA is numbered according to Ensembl transcript ID ENST00000378043, in which +1 is the A of the translation start codon.

**Table 1:** Primer and annealing conditions for bestrophin-1 (*BEST1*) sequencing.

**Annealing temperature (°C)**	**Additional information**	**Primer name**	**Sequence 5′-3′**
		Exon 2F	CACCTGCTGCAGCCCACTGCC
61		Exon 2R	CTTGTAGTGAACTGGTACACTGGCC
		Exon 3F	GGACAGTCTCAGCCATCTCCTCG
59		Exon 3R	GCAGCTCCTCGTGATCCTCCCCTGG
		Exon 4F	CTAGGCCCGCTCGCAGCAGAAAGC
60	10% DMSO	Exon 4R	CTTCCATTCCTGCCGCGCCCATCTC
		Exon 5/6F	CATCCCTTCTGCAGGTTCTC
59		Exon 5/6R	CTTGGTCCTTCTAGCCTCAGTTTC
		Exon 7F	CTGGAGCATCCTGATTTCAGGGTTC
59		Exon 7R	CTCTGGCCATGCCTCCAGC
		Exon 8/9F	GCTGGCTTTGAGGAGTTCTGCCTG
59		Exon 8/9R	GTGCTATTCTAAGTTCCTAGGCAG
		Exon 10F	GTAAGGGAGAAGTAAGGCCAGGTG
59		Exon 10 R	GTAGGTCCAGTGTGCTCTGGCAG
		Exon 11F	GAAGGGACCTTCCATACTTATG
59		Exon 11R	CATTAAAGGCTGAAGTAGTCTGGG

Haplotype analysis was performed in one family by genotyping four microsatellite markers that flank the *BEST1* gene: D11S935, D11S4102, D11S987, and D11S4162, available on the ABI linkage mapping set v.2.5-MD10. PCR was performed on a thermal cycler in a total volume of 9 μl containing 100 ng of genomic DNA, 0.2 μl of fluorescently labeled microsatellite markers (ABI, Foster City, CA), and equal volumes of ABsolute^TM^ QPCR-mix (ABgene; Thermo Scientific, Epsom, UK). Reactions were performed with the standard thermocycling profile for all markers. This consisted of an initial denaturation (95 °C) for 15 min followed by 35 cycles of 94 °C for 60 s, 57 °C for 60 s, and 72 °C for 60 s, with a final extension step of 72 °C for 12 min. One microliter of each PCR product was diluted in 12 μl of the HiDi formamide containing 2.5 μl of GeneScan^TM^-500 LIZ ® fluorescent dye (Applied Biosystems). The samples were denatured for 5 min at 95 °C, snap-frozen on ice for 5 min, and centrifuged before loading onto the ABI Prism ® 3730 DNA Analyzer. The GeneMapper version 4.0 program (Applied Biosystems) was used to score the genotypes.

All participating individuals consented to the investigation as part of an ethically approved research project that was performed in accordance with the tenets of the Declaration of Helsinki.

## Results

The EOG Arden ratios in the normal subjects ranged from 180% to 435% (mean 260%, standard deviation [SD] 50%, n=30). An abnormal EOG typical for Best disease was defined as an Arden ratio of <120% [28,29], while 155%–185% was considered borderline or equivocal, and >185% clearly normal [30–32]. The upper limits of normal timing (mean±2.5 SD) were 15 min for the dark trough and 12 min for the light peak.

### Proband 1 and family members

**Figure 1:**
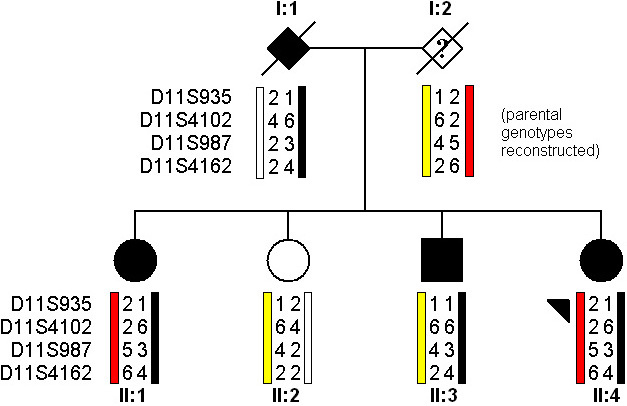
Pedigree structure and haplotype analysis of family 1. The diseased allele is indicated in black, while the haplotypes carried by the unaffected parent are indicated red and yellow.

**Figure 2:**
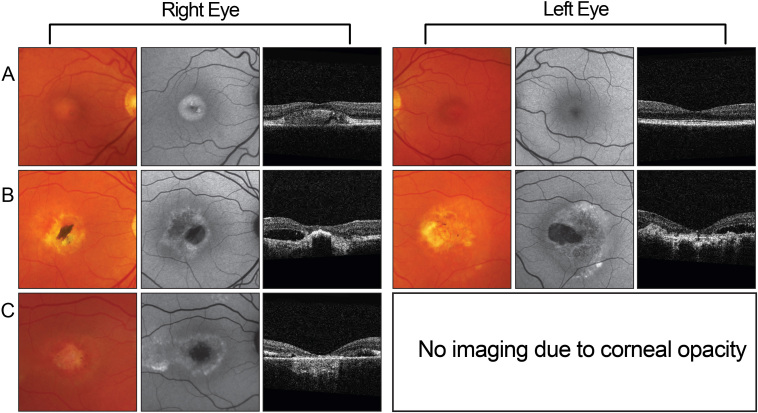
Fundus signs of affected siblings in family 1. Fundus photographs, autofluorescence, and posterior segment optical coherence tomography (OCT) images of family 1. **A**: Individual II:1 (affected sister) had a completely normal left posterior segment examination but significant retinal pigment epithelium (RPE) layer thickening on the right. **B**: Individual II:3 (affected brother) had disruption of the inner segment: outer segment interface. **C**: Individual II:4 (proband) had exudative vitelliform lesions, demonstrated in the right eye.

A 42-year-old white female (II:4) with bilateral macular scarring characteristic of Best disease was reviewed with her three older siblings. Figure 1 shows the pedigree structure. The family was of white English ancestry with no history of consanguinity. Both parents were deceased, and the only available family history was “poor sight” in the paternal grandmother. Two of the siblings of II:4 (II:1 and II:3) had clinical findings compatible with Best disease. Figure 2 shows the fundus signs of the three affected individuals. The older affected sister, II:1 (Figure 2), had asymmetric disease, with a normal left fundus appearance and macular scarring in the right eye. Their affected brother, II:3 (Figure 2), had bilateral disease, and showed a fundus appearance similar to that of the proband (Figure 2). Autofluorescence imaging demonstrated an increase in lipofuscin accumulation around the macular region with no scarring in the periphery. Posterior segment OCT imaging showed cystoid macular lesions, with thickening (hypertrophy and disruption) of the layer between RPE and the inner segment/ outer segment interface. Their unaffected sister (II:2) had normal fundus examinations.

**Figure 3:**
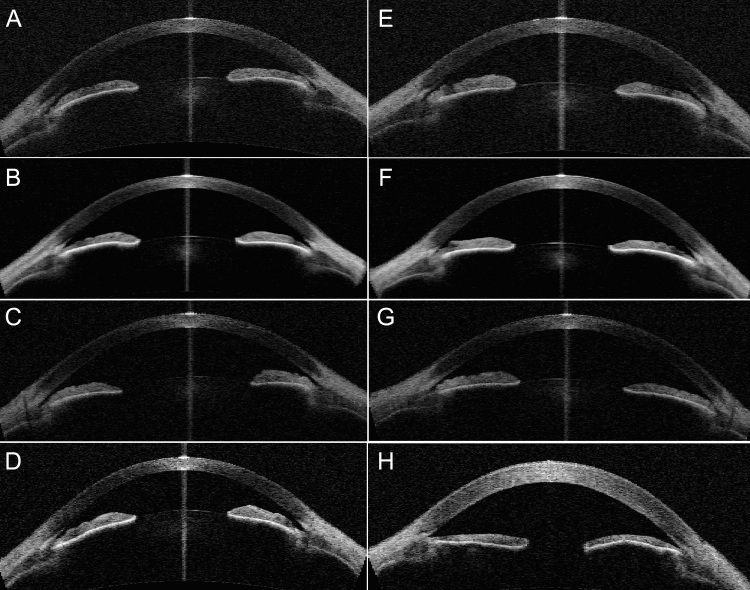
Anterior segment optical coherence tomography findings for family 1. **A**-**D**: Right eyes of individuals II:1 to II:4 respectively. **E**-**H**: Left eyes of individuals II:1 to II:4 respectively. The unaffected sister (II:2) shown in row 2 had closed angles on anterior segment (AS)-OCT. Slit openings were observed for individuals II:1 and II:3 on AS-OCT but their angles were closed on gonioscopy. The proband shown in row 4 had the shallowest anterior chamber depths. **H**: This shows a thickened cornea from aphakia and corneal decompensation from previous surgery in the proband.

A summary of the ocular characteristics is shown in Table 2. All four siblings had short axial biometry, but only the individuals affected with Best disease were found to be hypermetropic. The proband also had evidence of advanced secondary angle-closure glaucoma (ACG). Pupil-block angle closure was suspected on anterior segment OCT examination of the siblings (Figure 3), and was confirmed on dark-room gonioscopy where the convex iris configuration and iridotrabecular contact in more than 180° of the angle circumference was observed for all four siblings. Both sisters and the brother of the proband underwent laser iridotomies. Although the proband (II:4) was the youngest sibling, she had uncontrolled ACG, requiring multiple filtration and seton surgery that eventually led to corneal endothelial loss and significant corneal opacity (OS).

**Table 2:** Clinical and biometric characteristics.

**Individuals**	**BCVA**		**Refraction**		**IOP**		**ACD**		**AL**		**CDR**		**Fundus**	
** **	**OD**	**OS**	**OD**	**OS**	**OD**	**OS**	**OD**	**OS**	**OD**	**OS**	**OD**	**OS**	**OD**	**OS**
**Family 1**														
Affected sister (II:1)	6/60	6/9	+4.00	+4.00	18	14	2.52	2.43	20.46	20.84	0.3	0.3	SRF	Normal
Unaffected sister (II:2)	6/6	6/6	plano	plano	14	13	2.66	2.74	21.98	21.93	0.3	0.3	Normal	Normal
Affected brother (II:3)	6/36	6/36	+0.75	+0.75	19	20	2.56	2.64	22.54	22.6	0.3	0.3	VL, SRF	VL, SRF
Proband 1 (II:4)	6/18	CF	+3.50	+3.50	38	24	2.2	2.06	20.7	21.63	0.5	0.3	VL, SRF	VL, SRF
**Family 2**														
Proband 2	6/12	6/12	+3.00	+2.00	15	14	2.83	2.85	21.25	21.42	0.2	0.3	VL, SRF	VL, SRF

Abbreviations: BCVA represents best corrected visual acuity; IOP represents intraocular pressure; ACD represents anterior chamber depth; AL represents axial length; CDR represents cup: disc ratio; OD represents right eye; OS represents left eye; VL represents vitelliform lesion; SRF represents subretinal fluid.

**Figure 4:**
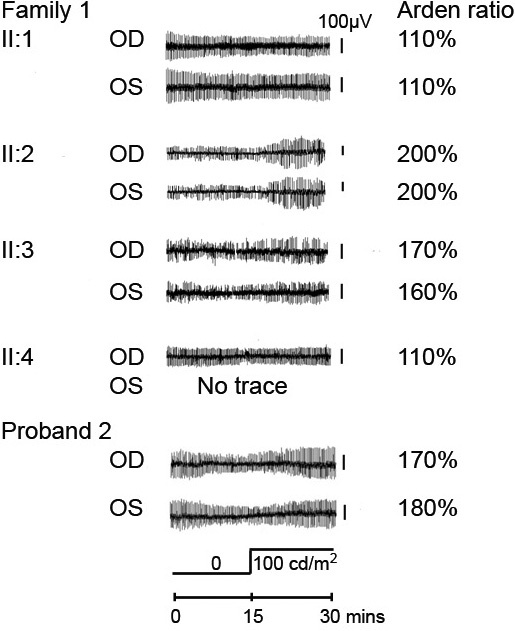
Electrooculography tracings. There is inter- and intrafamilial variability in the electrooculographic (EOG) findings. Individual II:2 is the only unaffected subject, with no clinical evidence of Best disease, and clearly normal EOG light rises. Both individuals II:3 and proband 2 demonstrate near-normal EOG light rises, which is atypical for Best disease.

Electrophysiological testing showed normal electroretinograms. EOGs showed minimal dark trough and light rise in the proband (II:4) and her affected sister (II:1; Figure 4). The affected brother (II:3) showed a borderline EOG light rise of 170% and 160% in the right and left eyes (Figure 4). His unaffected sister (II:2) had an EOG light rise of 200% bilaterally. Patient II:3 had a slight delay of the light peak at 14 min, but not the dark trough at 10 min. The other two affected siblings had normal dark trough and light peak timings.

**Figure 5:**
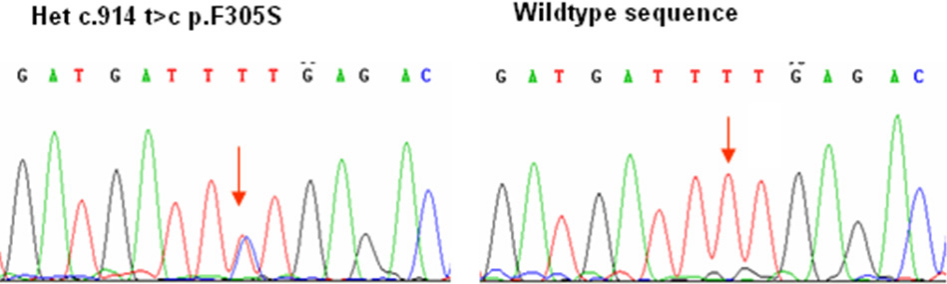
Genotype sequences for family 1. The sequence shown on the left was shared by all three affected siblings, while their unaffected sister had the wild-type sequence shown on the right.

Bidirectional Sanger sequencing of *BEST1* in the three affected siblings revealed a base substitution in the coding region (exon 8), c.914T>C, p.Phe305Ser, but no other variants (Figure 5). The p.Phe305Ser mutation has previously been described as segregating in a dominant fashion in another family with Best disease [1]. The unaffected sibling from this family was wild type at this allele. The haplotype carrying the mutation is illustrated in black (Figure 1). The affected brother (II:3) with a good EOG light rise had the same nondisease haplotype (yellow) as his unaffected sister (II:2). Neither parent was available for genetic analysis; however, the markers were informative enough to infer that the affected brother, II:3, had inherited a different allele from the noncarrier parent (I:2) compared to the other two siblings, II:1 and II:4 (red). See Figure 1.

### Proband 2

**Figure 6:**
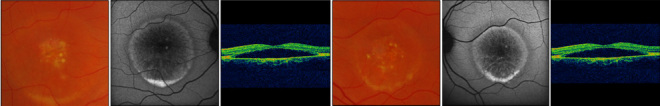
Fundus signs of proband. Color photographs and posterior segment optical coherence tomography of proband 2. The color photographs show significant subretinal fluid in both eyes under the central vitelliform lesions, confirmed on optical coherence tomography.

An unrelated 53-year-old white female from the UK presented to the clinic with presumed Best disease after deterioration of her vision following a viral illness. She had been diagnosed with macular dystrophy at the age of 40 years. There was a family history of poor sight in her mother and maternal grandfather, but no clinical details were known. No other family members were available for clinical examination or genetic testing. Her phenotypic characteristics are presented in Table 2. Fundus and OCT examinations showed significant accumulation of subretinal fluid and central vitelliform lesions (Figure 6). Her refraction and AL readings fell within the range of all four siblings from family 1, but she had deeper ACDs of 2.83/2.85mm OD/OS, and open angles on gonioscopy. Her EOG showed a normal dark trough with a light rise of 170% right eye and 180% left eye (Figure 4). Dark trough and light peak timings were normal. Bidirectional Sanger sequencing of *BEST1* revealed the patient to be heterozygous for the same missense variant in *BEST1* as that present in family 1 (c.914T>C, p.Phe305Ser).

## Discussion

This report addresses the clinical, electrophysiological, and genetic findings in two families with dominant Best disease caused by the same p.Phe305Ser mutation. Very few reports of *BEST1* mutation with normal or equivocal EOGs have appeared [12,33–35]. However, each of the two families herein reported contains one individual with an EOG light rise atypical for the disorder.

Hyperopia of greater than +3.00 diopters is a recognized a feature of Best disease [28]. All affected patients were hyperopic; only the unaffected sister of proband 1 had a normal refraction (Table 2) with a short AL (21.98 mm), similar to all the affected subjects (range: 20.46–22.60 mm). Short axial length is a risk factor for developing angle closure, shared by first degree relatives [36]. Although there are relatively few reports of Best disease and ACG [37–39], angle closure was present in all four siblings of family 1. In other bestrophinopathies, ACG is relatively common, affecting up to 50% of cases with ADVIRC [3–7] or ARB [8–10]. Proband 2 did not have angle closure, and this may be explained by her deeper ACD (Table 2).

Also of note is the completely normal left fundus of patient II:1. While marked fundus asymmetry can rarely occur in patients with Best disease [40,41], Testa et al. [35] reported an Italian family with one of three affected individuals having a completely normal fundus in one eye. That Italian family was one of the few reports of a normal EOG in Best disease; they carried a heterozygous mutation of p.Phe305Leu, the same amino acid as in this report. A normal fundus appearance in both eyes of one mutation-positive subject has been described [42] (p.Arg218Cys mutation). The Arden ratio was 175% in one eye, and 150% in the other. This subject’s affected daughter and grandchildren had findings typical of Best disease.

An abnormal Arden ratio of <120% is a characteristic finding in Best disease [28,39], with 155%–185% considered borderline [30–32]. Both patient II:3 and proband 2 had values between 160% and 180%, which is atypically high for Best disease, and which did not occur in other affected family members who had expectedly severe EOG abnormality. This inter- and intrafamilial difference in EOG findings suggests varying degrees of RPE dysfunction. A summary of the clinical and electrophysiological differences between disorders caused by *BEST1* mutations is shown in Table 3.

**Table 3:** Clinical differences between disorders caused by bestrophin-1 (*BEST1*) mutations.

**Clinical signs**	**Best disease** **[**28,33-35,38,39**]**	**ARB [**8-10**]**	**ADVIRC [**3-7**]**	**Concentric RP [**13**]**	**AVMD [**11,12**]**
**Mode of inheritance**	Autosomal dominant	Autosomal recessive	Autosomal dominant	Autosomal dominant	Autosomal dominant
**Fundus lesion**	Central macula vitelliform lesions	Diffuse RPE disturbance and dispersed punctate flecks, subretinal fluid may fluctuate	Peripheral circumferential pigmentation, and peripapillary chorioretinal atrophy	Foveal deposits, prone to serous retinal detachments. Intraretinal bone spicule pigmentation - often showing an abrupt change between normal and abnormal retina	Milder phenotype characterized by RPE atrophy; small drusen-like deposits in the paracentral region
**Lens changes**	Not known	Not known	Spherophakia and cataract	Cataract	Nil
**Refraction**	One third ≥ +3.00 DS	Range from +0.25 to +4.75 DS	−2.50 DS to +15.00 DS depending on axial biometry / presence of posterior staphyloma	Limited data suggest marked interfamilial variability from high hyperopia to high myopia	Unknown
**Other features**	ACG	ACG	ACG, microcornea, iris dysgenesis, and posterior staphyloma	Nil	Nil
**EOG findings of relevance to this study**	Normal/ near-normal EOGs reported for p.F305S/L, p.A243V, p.I295del	No cases of affected individuals with normal EOGs to date	Marked inter and intra-familial variability for EOG findings	EOGs not tested	Mildly reduced EOG for p.A243V

Abbreviations: ARB represents autosomal recessive bestrophinopathy; ADVIRC represents autosomal dominant vitreoretinochoroidopathy; RP represents retinitis pigmentosa; AVMD represents adult vitelliform macular dystrophy; RPE represents retinal pigment epithelium; DS represents diopters sphere; ACG represents angle-closure glaucoma; EOG represents electrooculography.

Previously described *BEST1* mutations associated with preserved EOG include: p.Phe305Leu [35], p.Asp243Val [12,34], and p.Ile295del [38]. Whole-cell patch clamp analysis in HEK-293 cells by Yu et al. has demonstrated that the p.Asp243Val mutation causes approximately a 90% reduction in chloride-specific currents generated by bestrophin-1 [43]. The researchers also showed that the p.Ile295del mutation completely abolishes the chloride channel activity of bestrophin-1 [44]. The 305 amino acid position lies within the C-terminus of *BEST1*, one of three mutational hotspots [45]. This is a highly conserved region of protein sequence close to transmembrane 6, lying on the outer surface of the protein, and may be involved in charge-dependent ligand binding. How the p.Phe305Ser and p.Phe305Leu mutations affect the in vitro activity of bestrophin-1 is yet to be identified and warrants further investigation.

Currently unidentified genetic mechanisms such as a single nucleotide polymorphism or a small change in the expression of the wild-type allele in the surrounding sequence may help to mitigate the EOG light rise. Only one of the three affected siblings of family 1 demonstrated a near-normal EOG light rise in the present study. To test the hypothesis that the nondisease allele was mitigating the effect, microsatellite markers flanking the *BEST1* gene were genotyped. The results suggested that the nondisease allele from the noncarrier parent might determine the difference in EOG. This would be consistent with reduced penetrance of the p.Phe305Ser mutation in abolishing the EOG light rise in some cases. However, the probability of this occurring by chance alone is 25%.

The role of bestrophin-1 in ocular development, and thus its role in the development of ACG, has not been elucidated. Anterior segment changes at the cornea and lens are present in ADVIRC, and extremes of axial biometry (range: 16–25 mm) can occur, depending on whether a posterior staphyloma is present [5]. Typical fundus signs (Table 3), with hyperopia and short axial biometry are almost always present in patients with Best disease [28,37,39] and ARB [8–10]. This association between *BEST1* mutations and ACG is important for clinicians.

In conclusion, this report details unusual phenotypic characteristics in two families with Best disease, both carrying the same missense p.Phe305Ser mutation. They include mutation-positive patients with a relatively well preserved EOG light rise; the presence of angle closure in all affected siblings of family 1; and a completely normal fundus in one eye of one of these affected patients. An effect of the nondisease allele is likely to contribute to the reduced penetrance of the Best disease phenotype, at least for the EOG light rise. Given the significant risk of ACG, assessment of angle-closure risk is a necessary consideration in all types of *BEST1* related disease.

## References

[1] Marquardt A, Stohr H, Passmore LA, Kramer F, Rivera A, Weber BH (1998). Mutations in a novel gene, VMD2, encoding a protein of unknown properties cause juvenile-onset vitelliform macular dystrophy (Best's disease).. Hum Mol Genet.

[2] Petrukhin K, Koisti MJ, Bakall B, Li W, Xie G, Marknell T, Sandgren O, Forsman K, Holmgren G, Andreasson S, Vujic M, Bergen AA, McGarty-Dugan V, Figueroa D, Austin CP, Metzker ML, Caskey CT, Wadelius C (1998). Identification of the gene responsible for Best macular dystrophy.. Nat Genet.

[3] Burgess R, MacLaren RE, Davidson AE, Urquhart JE, Holder GE, Robson AG, Moore AT, Keefe RO, Black GC, Manson FD (2009). ADVIRC is caused by distinct mutations in BEST1 that alter pre-mRNA splicing.. J Med Genet.

[4] Lafaut BA, Loeys B, Leroy BP, Spileers W, De LJJ, Kestelyn P (2001). Clinical and electrophysiological findings in autosomal dominant vitreoretinochoroidopathy: report of a new pedigree.. Graefes Arch Clin Exp Ophthalmol.

[5] Reddy MA, Francis PJ, Berry V, Bradshaw K, Patel RJ, Maher ER, Kumar R, Bhattacharya SS, Moore AT (2003). A clinical and molecular genetic study of a rare dominantly inherited syndrome (MRCS) comprising of microcornea, rod-cone dystrophy, cataract, and posterior staphyloma.. Br J Ophthalmol.

[6] Vincent A, McAlister C, Vandenhoven C, Heon E (2011). BEST1-related autosomal dominant vitreoretinochoroidopathy: a degenerative disease with a range of developmental ocular anomalies.. Eye (Lond).

[7] Yardley J, Leroy BP, Hart-Holden N, Lafaut BA, Loeys B, Messiaen LM, Perveen R, Reddy MA, Bhattacharya SS, Traboulsi E, Baralle D, De Laey JJ, Puech B, Kestelyn P, Moore AT, Manson FD, Black GC (2004). Mutations of VMD2 splicing regulators cause nanophthalmos and autosomal dominant vitreoretinochoroidopathy (ADVIRC).. Invest Ophthalmol Vis Sci.

[8] Burgess R, Millar ID, Leroy BP, Urquhart JE, Fearon IM, De Baere E, Brown PD, Robson AG, Wright GA, Kestelyn P, Holder GE, Webster AR, Manson FD, Black GC (2008). Biallelic mutation of BEST1 causes a distinct retinopathy in humans.. Am J Hum Genet.

[9] Davidson AE, Sergouniotis PI, Burgess-Mullan R, Hart-Holden N, Low S, Foster PJ, Manson FD, Black GC, Webster AR (2010). A synonymous codon variant in two patients with autosomal recessive bestrophinopathy alters in vitro splicing of BEST1.. Mol Vis.

[10] Wittström E, Ekvall S, Schatz P, Bondeson ML, Ponjavic V, Andreasson S (2011). Morphological and functional changes in multifocal vitelliform retinopathy and biallelic mutations in BEST1.. Ophthalmic Genet.

[11] Allikmets R, Seddon JM, Bernstein PS, Hutchinson A, Atkinson A, Sharma S, Gerrard B, Li W, Metzker ML, Wadelius C, Caskey CT, Dean M, Petrukhin K (1999). Evaluation of the Best disease gene in patients with age-related macular degeneration and other maculopathies.. Hum Genet.

[12] Krämer F, White K, Pauleikhoff D, Gehrig A, Passmore L, Rivera A, Rudolph G, Kellner U, Andrassi M, Lorenz B, Rohrschneider K, Blankenagel A, Jurklies B, Schilling H, Schütt F, Holz FG, Weber BH (2000). Mutations in the VMD2 gene are associated with juvenile-onset vitelliform macular dystrophy (Best disease) and adult vitelliform macular dystrophy but not age-related macular degeneration.. Eur J Hum Genet.

[13] Davidson AE, Millar ID, Urquhart JE, Burgess-Mullan R, Shweikh Y, Parry N, O'Sullivan J, Maher GJ, McKibbin M, Downes SM, Lotery AJ, Jacobson SG, Brown PD, Black GC, Manson FD (2009). Missense mutations in a retinal pigment epithelium protein, bestrophin-1, cause retinitis pigmentosa.. Am J Hum Genet.

[14] Deutman AF (1969). Electro-oculography in families with vitelliform dystrophy of the fovea. Detection of the carrier state.. Arch Ophthalmol.

[15] Bakall B, Marmorstein LY, Hoppe G, Peachey NS, Wadelius C, Marmorstein AD (2003). Expression and localization of bestrophin during normal mouse development.. Invest Ophthalmol Vis Sci.

[16] Guziewicz KE, Zangerl B, Lindauer SJ, Mullins RF, Sandmeyer LS, Grahn BH, Stone EM, Acland GM, Aguirre GD (2007). Bestrophin gene mutations cause canine multifocal retinopathy: a novel animal model for best disease.. Invest Ophthalmol Vis Sci.

[17] Marmorstein AD, Marmorstein LY, Rayborn M, Wang X, Hollyfield JG, Petrukhin K (2000). Bestrophin, the product of the Best vitelliform macular dystrophy gene (VMD2), localizes to the basolateral plasma membrane of the retinal pigment epithelium.. Proc Natl Acad Sci USA.

[18] Mullins RF, Kuehn MH, Faidley EA, Syed NA, Stone EM (2007). Differential macular and peripheral expression of bestrophin in human eyes and its implication for best disease.. Invest Ophthalmol Vis Sci.

[19] Gallemore RP, Hughes BA, Miller SS (1997). Retinal pigment epithelial transport mechanisms and their contributions to the electroretinogram.. Prog Retin Eye Res.

[20] Sun H, Tsunenari T, Yau KW, Nathans J (2002). The vitelliform macular dystrophy protein defines a new family of chloride channels.. Proc Natl Acad Sci USA.

[21] Marmorstein AD, Cross HE, Peachey NS (2009). Functional roles of bestrophins in ocular epithelia.. Prog Retin Eye Res.

[22] Marmorstein LY, Wu J, McLaughlin P, Yocom J, Karl MO, Neussert R, Wimmers S, Stanton JB, Gregg RG, Strauss O, Peachey NS, Marmorstein AD (2006). The light peak of the electroretinogram is dependent on voltage-gated calcium channels and antagonized by bestrophin (best-1).. J Gen Physiol.

[23] Rosenthal R, Bakall B, Kinnick T, Peachey N, Wimmers S, Wadelius C, Marmorstein A, Strauss O (2006). Expression of bestrophin-1, the product of the VMD2 gene, modulates voltage-dependent Ca2+ channels in retinal pigment epithelial cells.. FASEB J.

[24] Zhang Y, Stanton JB, Wu J, Yu K, Hartzell HC, Peachey NS, Marmorstein LY, Marmorstein AD (2010). Suppression of Ca2+ signalling in a mouse model of Best disease.. Hum Mol Genet.

[25] Brown M, Marmor M, Vaegan Zrenner E, Brigell M, Bach M (2006). ISCEV Standard for Clinical Electro-oculography (EOG) 2006.. Doc Ophthalmol.

[26] Holder GE, Brigell MG, Hawlina M, Meigen T, Vaegan Bach M (2007). ISCEV standard for clinical pattern electroretinography–2007 update.. Doc Ophthalmol.

[27] Marmor MF, Fulton AB, Holder GE, Miyake Y, Brigell M, Bach M (2009). ISCEV Standard for full-field clinical electroretinography (2008 update).. Doc Ophthalmol.

[28] Bard LA, Cross HE (1975). Genetic counseling of families with Best macular dystrophy.. Trans Sect Ophthalmol Am Acad Ophthalmol Otolaryngol.

[29] Cross HE, Bard L (1974). Electro-oculography in Best's macular dystrophy.. Am J Ophthalmol.

[30] Arden GB, Barrada A (1962). Analysis of the electro-oculograms of a series of normal subjects: role of the lens in the development of the standing potential.. Br J Ophthalmol.

[31] Arden GB, Barrada A, Kelsey JH (1962). New clinical test of retinal function based upon the standing potential of the eye.. Br J Ophthalmol.

[32] Arden GB, Kelsey JH (1962). Changes produced by light in the standing potential of the human eye.. J Physiol.

[33] Krämer F, Mohr N, Kellner U, Rudolph G, Weber BH (2003). Ten novel mutations in VMD2 associated with Best macular dystrophy (BMD).. Hum Mutat.

[34] Pollack K, Kreuz FR, Pillunat LE (2005). Best's disease with normal EOG. Case report of familial macular dystrophy.. Ophthalmologe.

[35] Testa F, Rossi S, Passerini I, Sodi A, Di Iorio V, Interlandi E, Della Corte M, Menchini U, Rinaldi E, Torricelli F, Simonelli F (2008). A normal electro-oculography in a family affected by best disease with a novel spontaneous mutation of the BEST1 gene.. Br J Ophthalmol.

[36] Sihota R, Ghate D, Mohan S, Gupta V, Pandey RM, Dada T (2008). Study of biometric parameters in family members of primary angle closure glaucoma patients.. Eye (Lond).

[37] Sohn EH, Francis PJ, Duncan JL, Weleber RG, Saperstein DA, Farrell DF, Stone EM (2009). Phenotypic variability due to a novel Glu292Lys variation in exon 8 of the BEST1 gene causing best macular dystrophy.. Arch Ophthalmol.

[38] Wabbels B, Preising MN, Kretschmann U, Demmler A, Lorenz B (2006). Genotype-phenotype correlation and longitudinal course in ten families with Best vitelliform macular dystrophy.. Graefes Arch Clin Exp Ophthalmol.

[39] Wittström E, Ponjavic V, Bondeson ML, Andréasson S (2011). Anterior Segment Abnormalities and Angle-Closure Glaucoma in a Family with a Mutation in the BEST1 Gene and Best Vitelliform Macular Dystrophy.. Ophthalmic Genet.

[40] Maloney WF, Robertson DM, Duboff SM (1977). Hereditary vitelliform macular degeneration: variable fundus findings within a single pedigree.. Arch Ophthalmol.

[41] Ponjavic V, Eksandh L, Andréasson S, Sjöström K, Bakall B, Ingvast S, Wadelius C, Ehinger B (1999). Clinical expression of Best's vitelliform macular dystrophy in Swedish families with mutations in the bestrophin gene.. Ophthalmic Genet.

[42] Caldwell GM, Kakuk LE, Griesinger IB, Simpson SA, Nowak NJ, Small KW, Maumenee IH, Rosenfeld PJ, Sieving PA, Shows TB, Ayyagari R (1999). Bestrophin gene mutations in patients with Best vitelliform macular dystrophy.. Genomics.

[43] Yu K, Cui Y, Hartzell HC (2006). The bestrophin mutation A243V, linked to adult-onset vitelliform macular dystrophy, impairs its chloride channel function.. Invest Ophthalmol Vis Sci.

[44] Yu K, Qu Z, Cui Y, Hartzell HC (2007). Chloride channel activity of bestrophin mutants associated with mild or late-onset macular degeneration.. Invest Ophthalmol Vis Sci.

[45] Qu Z, Cheng W, Cui Y, Cui Y, Zheng J (2009). Human disease-causing mutations disrupt an N-C-terminal interaction and channel function of bestrophin 1.. J Biol Chem.

